# Validation of reference genes for quantitative real-time PCR in chemical exposed and at different age’s brackish water flea *Diaphanosoma celebensis*

**DOI:** 10.1038/s41598-021-03098-x

**Published:** 2021-12-08

**Authors:** Young-Mi Lee, Hayoung Cho, Ryeo-Ok Kim, Soyeon In, Se-Joo Kim, Eun-Ji Won

**Affiliations:** 1 Department of Biotechnology, College of Convergence Engineering, Sangmyung University, Seoul, 03016 Republic of Korea; 2 Genome Editing Research Center, Korea Research Institute Bioscience and Biotechnology, Daejeon, 34141 Republic of Korea; 3 Department of Marine Science and Convergent Technology, Hanyang University, Ansan, 15588 Republic of Korea; 4 Present Address: Division of Chemical Research, National Institute of Environmental Research, Hwangyeong-ro 42, Seo-gu, Incheon, 22689 Korea

**Keywords:** Molecular biology, Environmental sciences

## Abstract

Real-time quantitative reverse transcription polymerase chain reaction (qRT-PCR), a primary approach for evaluating gene expression, requires an appropriate normalization strategy to confirm relative gene expression levels by comparison, and rule out variations that might occur in analytical procedures. The best option is to use a reference gene whose expression level is stable across various experimental conditions to compare the mRNA levels of a target gene. However, there is limited information on how the reference gene is differentially expressed at different ages (growth) in small invertebrates with notable changes such as molting. In this study, expression profiles of nine candidate reference genes from the brackish water flea, *Diaphanosoma celebensis*, were evaluated under diverse exposure to toxicants and according to growth. As a result, four different algorithms showed similar stabilities of genes for chemical exposures in the case of limited conditions using the same developmental stage (*H2A* was stable, whereas *Act* was fairly unstable in adults), while the results according to age showed a significantly different pattern in suite of candidate reference genes. This affected the results of genes *EcRA* and *GST*, which are involved in development and detoxification mechanisms, respectively. Our finding is the first step towards establishing a standardized real-time qRT-PCR analysis of this environmentally important invertebrate that has potential for aquatic ecotoxicology, particularly in estuarine environments.

## Introduction

Environmental pollutants, such as heavy metals and persistent organic pollutants, have been of great concern because of their persistence in aquatic environments and harmful effects on aquatic organisms^1,2^. In order to manage pollutants in aquatic environments, the diagnosis of biological responses is considered to be a significant factor in identifying the pollution or for further actions such as setting the guidelines^3^. Gene expression in the cell is the first step in the biological response to environmental stressors, resulting in biochemical, morphological, and functional changes. Reverse transcription—polymerase chain reaction (RT-PCR) is a highly sensitive and reproducible method for investigating gene expression^4^. In recent decades, RT-PCR has become a leading technique with the advantages of high sensitivity, specificity, and repeatability in several studies conducted in the field, including aquatic environments^5^. However, since variations in the initial RNA amount and efficiency of cDNA synthesis can lead to errors in RT-PCR results, a normalization step using stable and constantly expressed genes is crucial^6^. Additionally, the range of impacts of environmental pollutants depends on several factors, including concentration and duration of exposure to chemicals, as well as the stage of development, especially in species with molting systems^7^.

In general, the expression value (Ct) of the target gene was normalized to that of the reference genes, called the housekeeping genes, to compensate for differences among RNA samples^8,9^. Commonly used reference genes include the *18S rRNA*, *β-actin*, and *GAPDH* genes^10–12^ in crustaceans (mainly large crustaceans). However, few studies have validated reference genes in small crustaceans^13,14^, although many concerns arise in selection of appropriate genes. For example, *GAPDH*, *EF-α*, and *18S rRNA*, the most popular genes still preferred in aquatic invertebrate for normalization, remain unvalidated^15^, and some genes suggested as reference genes can be modulated by different environmental stresses^16^, indicating that even housekeeping genes may be involved in pathways other than cellular homeostasis^17^. Thus no gene is truly stable in all cells under all experimental conditions. Thus, reliable reference genes should be selected prior to experiments, and it is recommended that at least two reference genes be employed to avoid incorrect results^18,19^.

In aquatic ecosystems, coastal areas are vulnerable to land pollution due to substances derived from industries, wastewater, farming, and tourism. Many studies have shown that aquatic organisms, whether in situ environments or cultured in the laboratory, can be used to evaluate pollution and to test ecotoxicological responses^20,21^. In particular, brackish water species have a wide range of adaptations to environmental physico-chemical conditions, such as salinity and temperature, and are considered suitable for monitoring and risk assessment of various pollutants originating from land and marine environments. The brackish water flea *Diaphanosoma celebensis*, that belongs to Crustacea, Branchiopoda, and Cladocera, is one of the species of interest to many scientists as potential non-model species for marine pollution^22–24^. They are widely distributed in tropical Asia at a wide range of salinity and temperature. In particular, *D. celebensis* has several advantages such as a short life cycle (about 4–5 days from hatching to reproductive maturity, and 2–3 weeks lifespan), filter-feeders that might be exposed to several pollutants both in water or particles, small body length, and easy culture under laboratory conditions. Most importantly, single-breeding makes genetically identified results in genomic information. This is a great advantage that can be used for transcriptomic research with high efficiency. In fact, in recent studies, the genome and transcriptome of *D. celebensis* were published^25,26^, leading to their potential use at the molecular level in marine ecotoxicology. However, studies on the validation of reference genes are not explored yet in this species.

In this study, we selected nine common housekeeping genes as candidate reference genes from previous studies^27–30^ and compared their stability in expressions to identify appropriate reference genes under conditions such as chemical exposure and age. For this, three representative chemicals, benzo[a]pyrene (B[a]P), bisphenol A (BPA), and mercury (Hg), were selected. In particular, we focused on their growth ages, as this experimental species has a molting process with different sensitivities to growth^31,32^. Therefore, we investigated the stability of the nine candidate reference genes under different ages (24-h, 4-day, 7-day, and 10-day) and measured the relative expression of target genes based on the conditions of reference genes.

## Results

### PCR amplification of nine candidate reference genes

Nine common reference genes in *D. celebensis*, alpha-tubulin (*Atb*), β-actin (*Act*), 18S ribosomal RNA (*18S*), glyceraldehyde-3-phosphate dehydrogenase (*GAPDH*), elongation factor 1-beta (*EF-1b*), ubiquitin conjugating enzyme (*UBC*), histone H2A (*H2A*), TATA-box binding protein (*TBP*), and succinate dehydrogenase (*SDH*) were selected as candidate references for the analysis of gene expression under stress conditions made by three representative chemicals (e.g., BPA, B[a]P, Hg), and different ages (24-h, 4-day, 7-day, and 10-day). The information of all nine candidates, such as their gene IDs, gene, size of the PCR products, and Tm values, is shown in Table 1. In general, the sizes of the qPCR products of all the tested genes ranged from 81 to 104 bp. The applicability of the primers designed for the qPCR amplification of these nine candidate genes was verified by RT-PCR and sequencing of the amplified products (Supplementary Information, Text S1). The unimodal melting curves indicate that the primers used in this study have high specificity (Text S1). Furthermore, the efficiency values ranged from 90.8 to 106.7% (Table 1). Therefore, our qRT-PCR results were confirmed to be valid and reliable.

**Table 1:** Summary of nine reference genes.

Gene symbol	Gene description	GenBank accession no	Primer sequences (5′-3′)	Amplicon size (bp)	Tm (°C)	Efficiency (%)
*Atb*	*Alpha-tubulin*	MH636293	GCATGATTTCCAACACGAC TACCAGTGAACGAAGGCT	97	55.2	93.92
					53.9	
*Act*	*Beta-actin*	MH636289	CAAGATTGTCGCTCCTCCTG CATCTGCTGGAAGGTGG	84	60.5	90.79
					59.5	
*18 s*	*18 s ribosomal RNA*	AF144210.1	TGGAAGGATTGACAGATTGA CTTAGTTGGTGGAGCGATTT	81	54.3	101.89
					56.4	
*GAPDH*	*Glyceraldehyde-3-phosphate dehydrogenase*	MH636290	AACTGTCGCCGCTGTTGA ATGGAATGTTCTTGGGGTCG	86	56.1	96.31
					58.4	
*EF-1b*	*Elongation factor 1-beta*	MH636295	CGGCTGTGTCGTTGAAGA GGCAATGTCCACACTCTG	94	56.1	106.67
					56.1	
*UBC*	*Ubiqutin Conjugating enzyme*	MH636294	CCTTTTGACGGACCCATAT TAGTCCAACAGCGAGCAATA	104	55.2	103.89
					56.4	
*H2A*	*Histone H2A*	MH636296	GAATACCTGGCTGCTGAAG CAATTGGAGATGACGGGG	90	56.1	101.35
					57.3	
*TBP*	*TATA-box binding protein*	MH636292	TTTCCTGGCTTAATTTACCGTA ATCTCTTGTCTGACTTTGGC	101	54.3	96.37
					56.4	
*SDH*	*Succinate dehydrogenase*	MH636291	CATCGAGTCTCAACAGAAGA GAGCTCAACCTTTCCAGTT	91	56.4	100.16
					55.2	

### Expression level of nine reference genes

As shown in the results and box and whisker plots (Tables 2, 3, and Fig. S1), the Ct value distribution of nine reference genes under different conditions varied from 8.7 to 28.3. The *18S* gene encoded the most abundant transcripts in *D. celebensis*, reaching the threshold fluorescence peak after approximately 10 cycles with the lowest median Ct value (Ct < 10, Tables 2, 3, and Fig. S1) compared to those of other candidate reference genes during qPCR amplification when the same amounts of total RNA (500 ng) were used as templates for reverse transcription reactions. The least abundant transcripts were *Atb*, which had a Ct value of 21 or higher (21.93 ± 0.38), for chemical treatment. In a test using different ages, UBC had the highest Ct mean values (25.80 ± 0.54). However, with regard to variations in Ct values, *Atb* genes showed significant differences between all ages of development (ANOVA, *p* < 0.05).

**Table 2:** Cycle threshold (Ct) values of nine putative reference genes in 12 mRNA at different conditions (three chemicals and four concentrations) from a monoculture of *Diaphanosoma celebensis* exposed to chemicals.

Gene	Ct value statistics						
	Min	Max	Max–Min	Mean	SD	SE	CV
*Atb*	21.18	22.48	1.30	21.93	0.38	0.06	0.02
*Act*	16.06	16.52	0.46	16.26	0.09	0.01	0.01
*18S*	8.70	9.61	0.91	9.19	0.21	0.03	0.02
*GAPDH*	17.00	18.27	1.27	17.32	0.23	0.03	0.01
*EF-1b*	17.34	18.39	1.05	17.80	0.20	0.03	0.01
*UBC*	21.40	22.23	0.83	21.82	0.20	0.03	0.01
*H2A*	18.26	18.98	0.72	18.49	0.17	0.02	0.01
*TBP*	20.85	21.65	0.8	21.21	0.20	0.03	0.01
*SDH*	18.60	19.54	0.94	19.00	0.19	0.03	0.01

Min., minimum; Max., maximum; SD, standard deviation; SE, standard error; CV, coefficient of variation.

**Table 3:** Cycle threshold (Ct) values of nine putative reference genes in 12 mRNA samples from a monoculture of *Diaphanosoma celebensis* at different ages (Min., minimum; Max., maximum; SD, standard deviation; SE, standard error; CV, coefficient of variation).

Gene	Ct value statistics						
	Min	Max	Max–Min	Mean	SD	SE	CV
*Atb*	18.85	28.27	9.42	23.82	3.41	0.57	0.14
*Act*	18.97	21.29	2.32	19.75	0.57	0.10	0.03
*18S*	9.59	13.43	3.84	11.18	1.14	0.19	0.10
*GAPDH*	19.41	21.79	2.38	20.31	0.63	0.11	0.03
*EF-1b*	21.51	24.23	2.72	22.20	0.69	0.12	0.03
*UBC*	24.99	26.80	1.81	25.80	0.54	0.09	0.02
*H2A*	22.22	23.86	1.64	22.88	0.45	0.08	0.02
*TBP*	24.21	26.85	2.64	25.39	0.90	0.15	0.04
*SDH*	22.00	24.12	2.12	22.90	0.46	0.08	0.02

### The stability of candidate reference genes under chemical exposures

**Figure 1:**
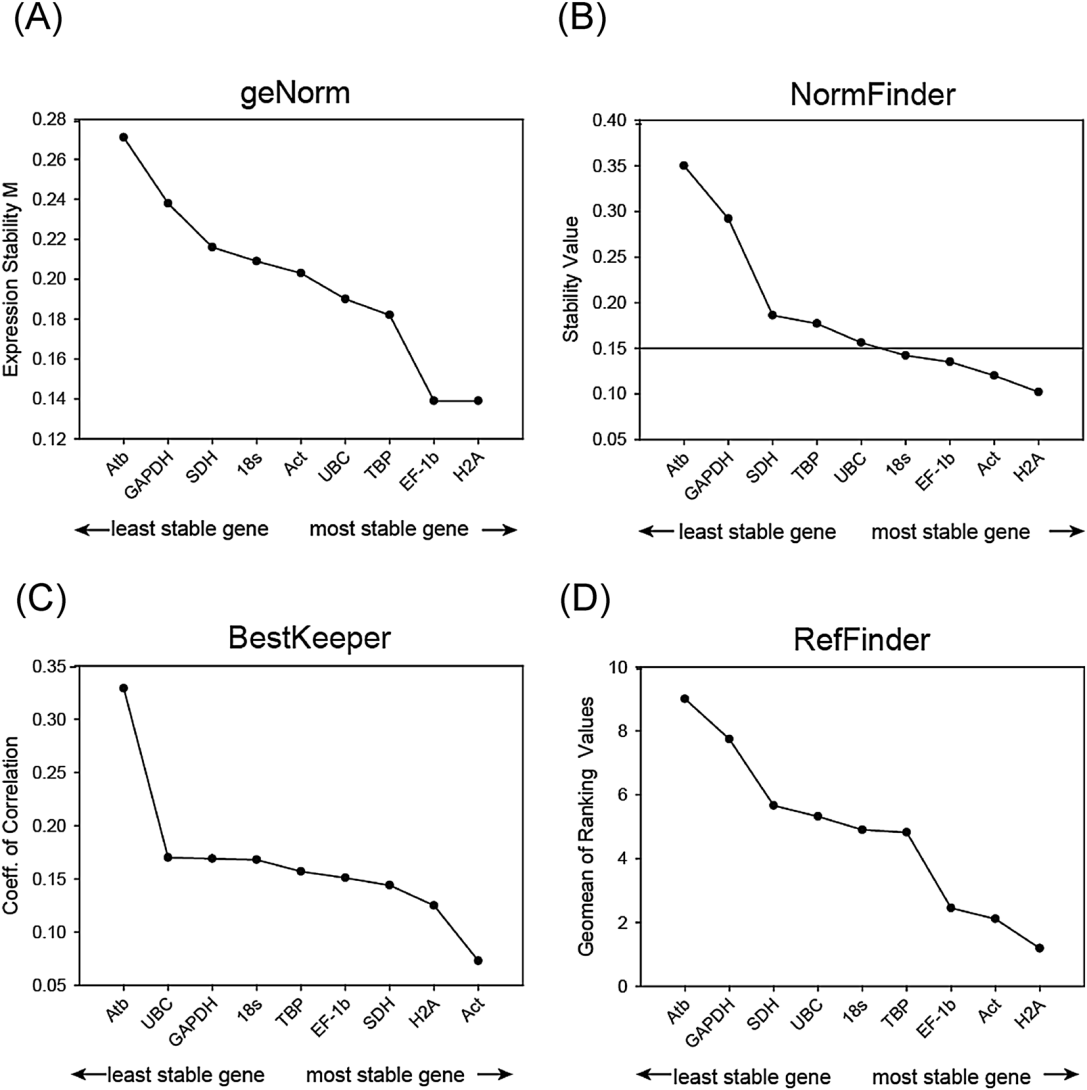
Expression stability of nine candidates for the normalization calculated by different algorithms for three different chemical (B[a]P, BPA, and Hg) exposures. Reference genes are shown in ascending order of expression stability. (**A**) geNorm (stability M) (**B**) NormFinder, (**C**) BestKeeper coefficient of correlation, and (**D**) RefFinder, a geomean of ranking values integrating other methods.

The stability of the reference genes showed different results according to the chemicals (Fig. 1, Fig. S2-S5). Based on the M values evaluated by geNorm, all genes and chemicals showed M values of less than 1, an acceptable value considering suitable reference genes for RT-qPCR normalization in all chemical exposure conditions (Fig. 1, Fig. S2). *Act* and *GAPDH* were the best reference genes for samples exposed to B[a]P (Fig. S2A) and BPA (Fig. S2B). Upon exposure to Hg, *UBC* and *TBP* were the most suitable reference genes (Fig. S2C). When considering all samples exposed to three different chemicals, the M values of all genes were stable in the following order: *H2A*, *EF-1b*, *UBC*, *TBP, Act*, *18S*, *SDH, GAPDH,* and *Atb* (Fig. 1).

NormFinder also showed different results depending on the sample group exposed to the chemical (Fig. S3). *Act*, *Atb*, and *TBP* were the most stable genes for exposure to B[a]P, BPA, and Hg, respectively (Fig. S3A-S3C). The results calculated by NormFinder were somewhat similar to those from geNorm in some representative genes in stability order for each chemical and whole samples (Fig. 1B).

For all cases, including each chemical and all samples, *Act* was the most stable reference gene in BestKeeper (Fig. 1C and Fig. S4). This method showed similar ranking results to other methods, but the order was observed to be different for Hg exposure (Fig S4C). Upon exposure to Hg, other methods (geNorm and NormFinder) showed that *TBP* and *UBC* were suitable, but only the BestKeeper showed *Act* and *H2A* as suitable candidates. This result is also the same in the case where all samples were gathered.

RefFinder, a web-based analysis tool, integrates three major computational programs, geNorm, NormFinder, and BestKeeper, and the comparative ΔCt method to comprehensively rank the tested candidate genes. This comprehensive method summarizes *H2A* and *Act* as potential reference genes for the chemical treatment of this species (Fig. 1D and Fig. S5).

Interestingly, in BPA exposure, *Atb*, which showed the most unstable results for the entire sample, was calculated as a stable reference gene as the first (NormFinder) or top ranking in several methods (third stable gene for BestKeeper, second stable gene for RefFinder) only for BPA exposure (Fig. S2-S5). When all three chemicals are considered together, either at the commonly used thresholds (1.5 for M-value for geNorm, SD lower than 1 for BestKeeper) or stability order (RefFinder), *H2A* and *Act* have similarly high stability.

### The stability of candidate reference genes under biotic conditions: different ages

**Figure 2:**
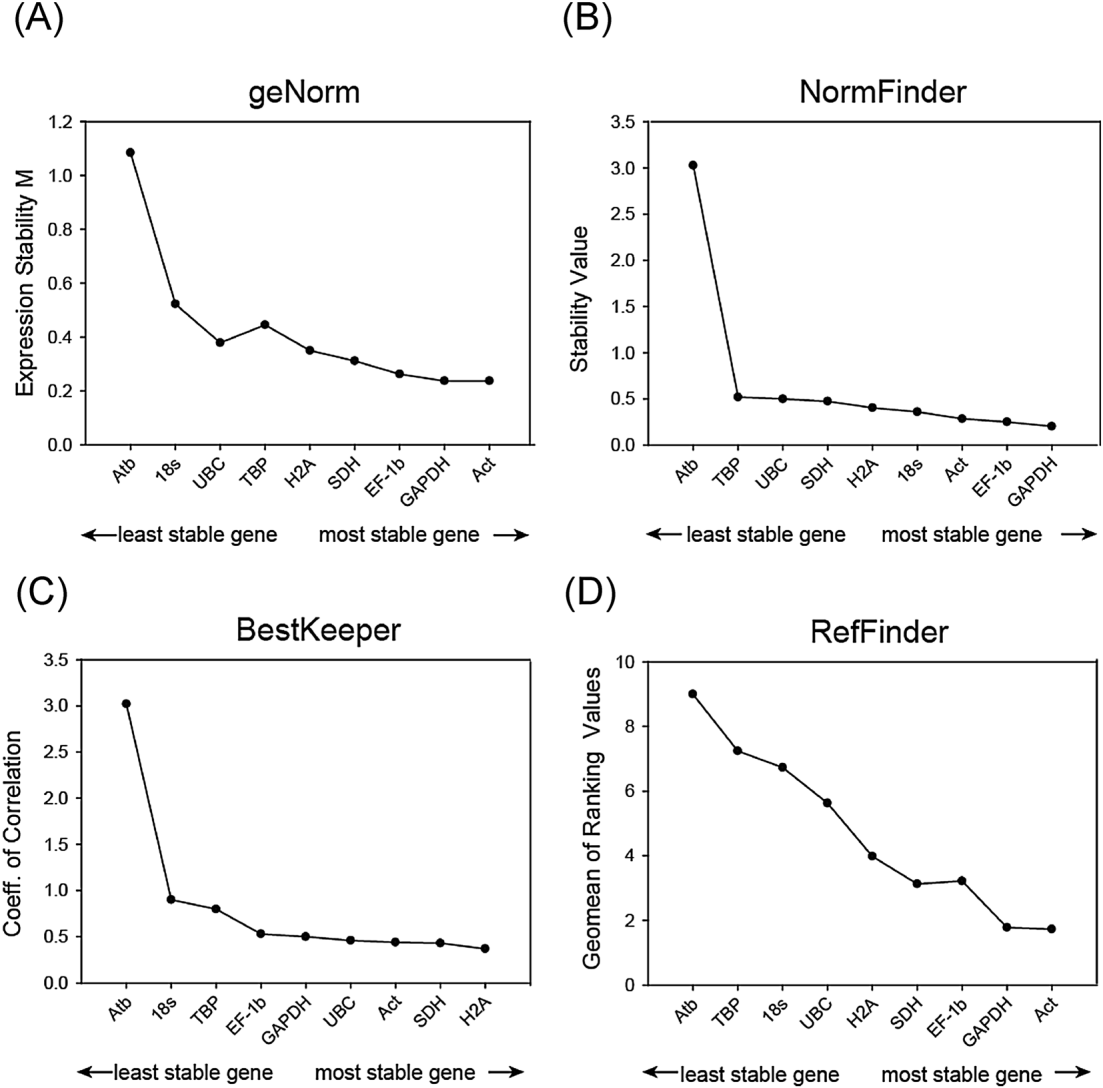
Assessment of nine putative reference genes of *Diaphanosoma celebensis* in different developmental ages (24 h, 4d, 7d, and 10 d) by four different algorithms (**A**) geNorm, (**B**) NormFinder, (**C**) BestKeeper, and (**D**) RefFinder. Reference genes are shown in ascending order of expression stability.

Each age group (24 h, 4 d, 7 d and 10 d post hatching) showed different stabilities for the nine reference genes of the brackish water flea *D. celebensis* (Fig. 2, Fig. S6-S9). Interestingly, individuals at post-24 h showed *TBP* to be the most unstable, whereas this gene was relatively stable at other ages of growth (days 4, 7, and 10). Even on day 4, the *TBP* gene was found to be the most stable in the NormFinder method (Fig. S7). All results showed that *Atb* was the most unstable, except at 24 h (Fig. 2 and Fig. S6-S9). In particular, the stability of *Atb* gene expression on day 4 was significantly different from that of the other genes. For example, in geNorm, only *Atb* had an M value greater than 1, and the next unstable gene, *18S*, was 0.5.

NormFinder and BestKeeper also showed that *Atb* was the most unstable gene, and also showed a large difference in stability value with the next unstable genes*, SDH* and *18S*, respectively (Fig. 2 and Fig S6-S8). NormFinder showed different results from geNorm in the order of stability order: *GAPDH*, *EF-1b*, *Act*, *18S*, *H2A*, *SDH*, *UBC*, *TBP*, and *Atb*, while the three most stable genes were the same. BestKeeper showed that *SDH* is the most stable gene when considering all age samples. The stable results of *18S* shown in geNorm and NormFinder do not apply to the seven-day individuals as coefficients in BestKeeper of *18S* over 1, the threshold of this algorithm. Finally, the integrated approach RefFinder indicates that *Act* is the most stable gene for covering all ages of *D. celebensis* (Fig. S8).

### Impact of reference genes on real-time qRT-PCR data analysis of target genes

**Figure 3:**
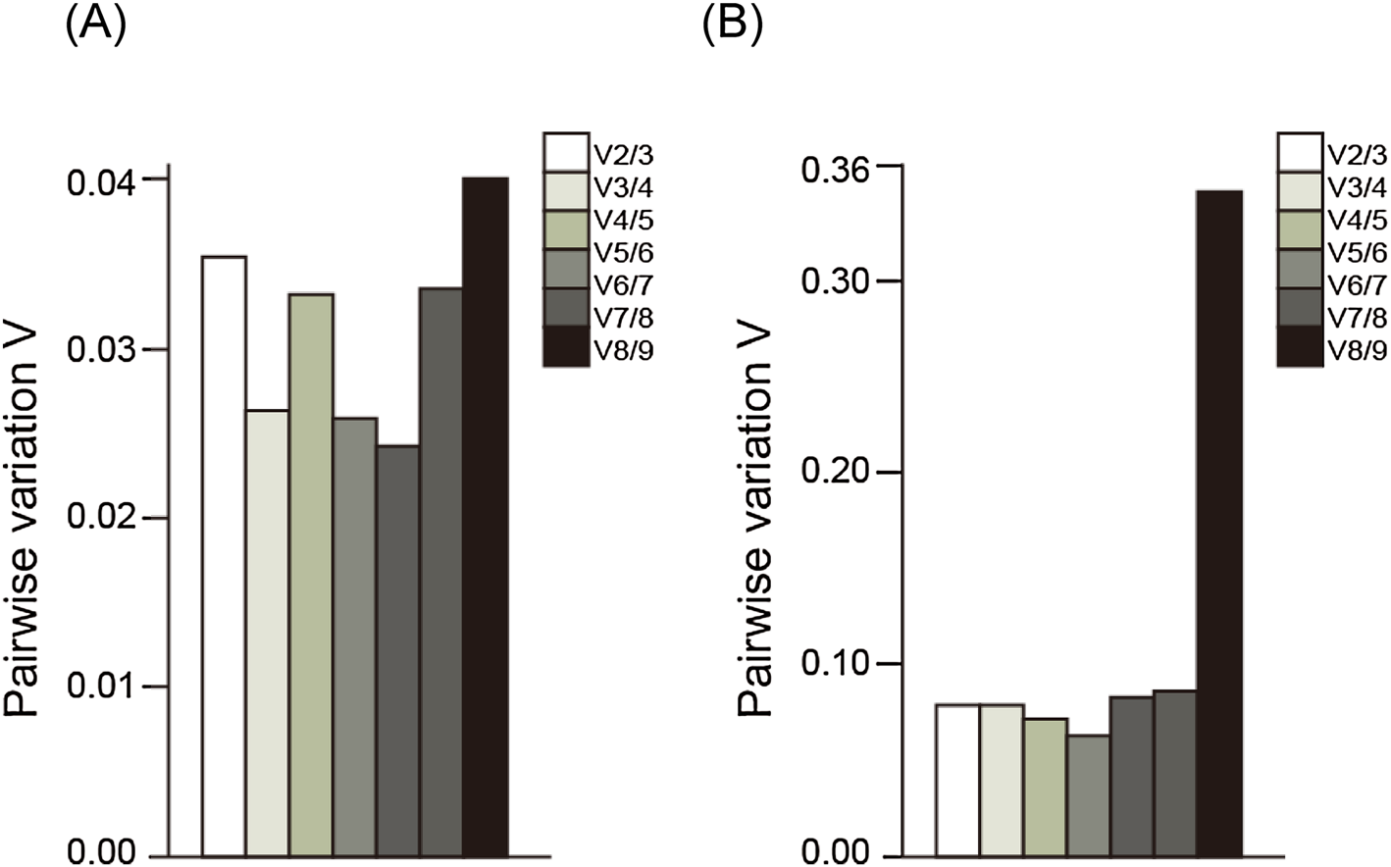
Pairwise variation for evaluating the optimal number of reference genes for accurate normalization (Vn/Vn + 1) in (**A**) chemical exposure and (**B**) ages. The line indicates the cut-off value (0.15), below which the inclusion of an additional reference gene is not required.

To validate the selected reference genes using algorithms, the expression of target genes was normalized and compared at different conditions. First, the pairwise variation (V) suggested that two reference genes are required for the normalization of gene expression levels (Fig. 3). In fact, all V values evaluated by geNorm were less than 0.15, which is the criterion for selecting a suitable reference gene number for the normalization of gene expression in chemical treatments. However, a test conducted using individuals of different ages showed that increasing the number of reference genes did not always increase reliability (Fig. 3B). For differential ages, *Act* and *GAPDH* were selected using RefFinder as stable reference genes.

**Figure 4:**
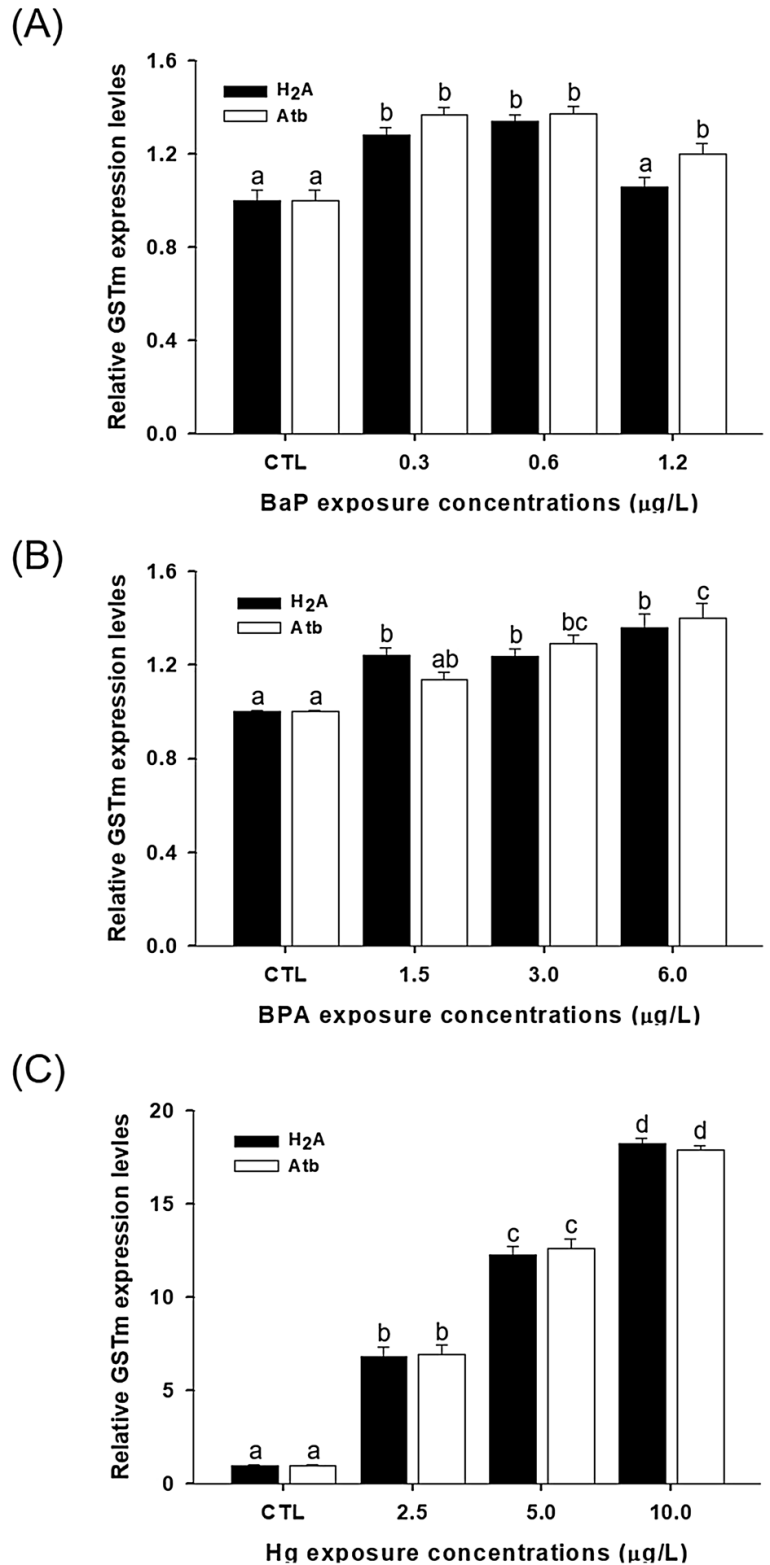
The relative expression level of *GSTmu* was determined using select reference genes, including the most (*H2A*) or least stable (*Atb*) reference genes, for normalization in the brackish water flea *D. celebensis* exposed to (**A**) B[a]P, (**B**) BPA, and (**C**) Hg. Different letters indicate significant differences between groups using different reference genes (ANOVA, Tukey’s post-hoc analysis, *p* < 0.05).

**Figure 5:**
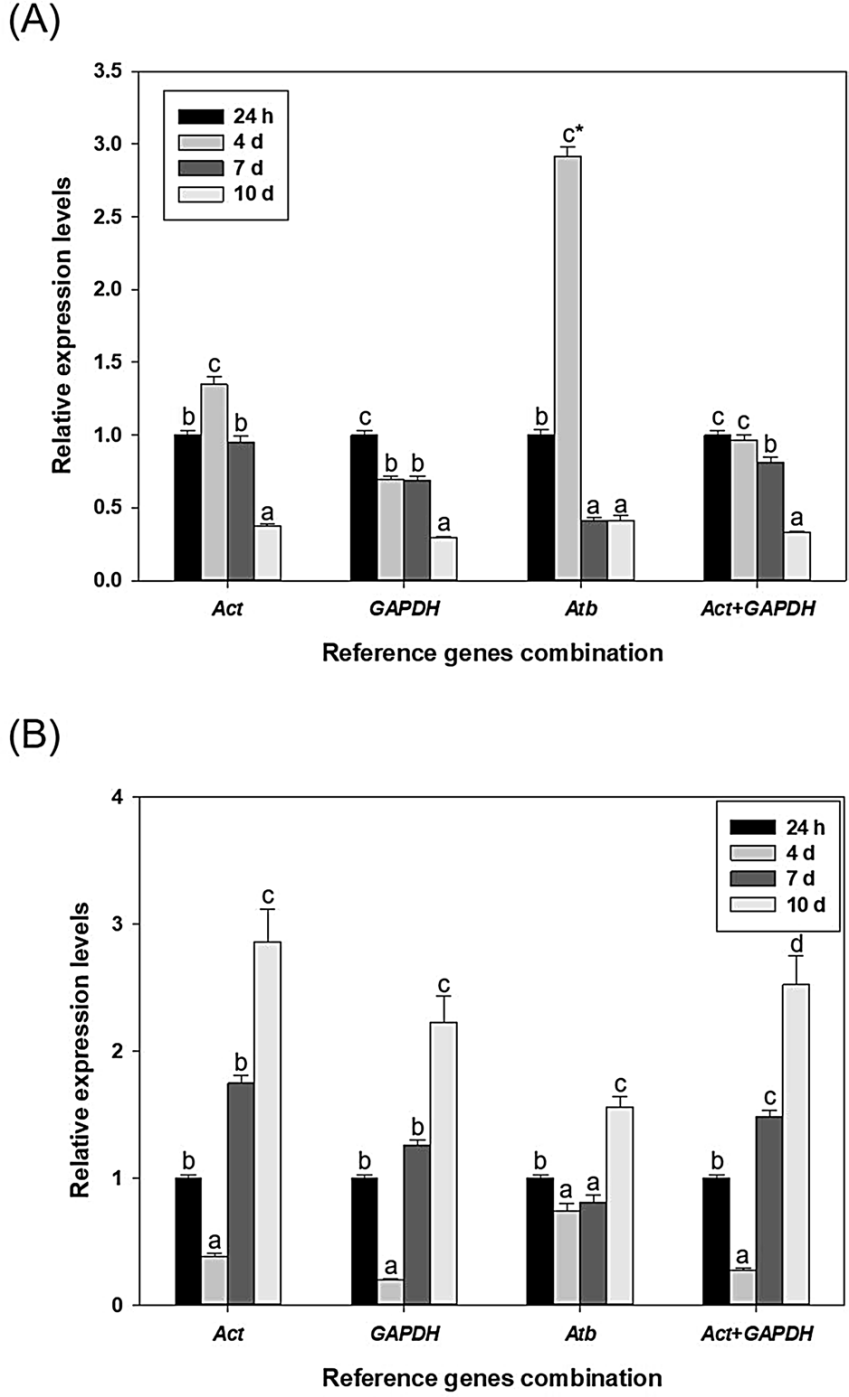
Relative quantification of (**A**) *GSTsigma* and (**B**) *EcRA* expression in *D. celebensis* at different ages, 24 h, 4 d, 7 d, and 10 d. The error bars represent the standard errors of the means of three biological replicates. Different letters and asterisks indicate significantly different expressions compared to the 24 h sample (ANOVA, *p* < 0.05) and differences under different normalizations of the reference gene at expression levels for the same age, respectively (t-test, *p* < 0.05).

The expression levels of *GSTmu* were compared between the most stable and unstable genes; however, no difference was found in the levels and patterns regardless of stable (*H2A*) or not (*Atb*) (Fig. 4). However, studies performed using *D. celebenesis* of different ages showed significantly different expression patterns of *GSTsigma* and *EcRA* depending on which reference gene was used for normalization (Fig. 5 and Fig. S10). In particular, the result using *Atb* as a reference gene showed superficially different expression patterns of target genes (highly expressed *GSTsigma* and *EcRA* at day 4) unlike other cases using stable reference genes (*GAPDH*, *Act*, and both genes).

## Discussion

With its rapid development, qRT-PCR has been widely used to evaluate the expression of target genes in recent years^33^. In particular, gene expressions in organisms collected in environments sometimes provide integrated information to assess environmental stress conditions without restricting the number of samples, times, and repeatability^34^. Recently, RNA transcriptomics has become an irreplaceable approach to measure the expression of the target gene in many fields based on biology in the rapid extraction of large-scale biological samples; however, these advantages, such as fast and mass processing, can cause inevitable errors. Thus, the so-called “housekeeping” genes as internal references have been attracting attention for several years and in all major organisms. Briefly, the absolute expression level of the target gene was calibrated based on that of the reference gene. This indicates that reliable validation of the reference gene is a pivotal step in the use of gene expression for further discussion using qRT-PCR. Furthermore, recent studies have reported that various physiological responses are affected by xenobiotic stress, including persistent organic pollutants and heavy metals^35,36^. The expression of specific genes associated with these physiological alterations shows biological repercussions and the status of the environment. This is the reason why the comprehensive evaluation of reference gene stability in response to diverse biotic and abiotic factors is a rising issue.

However, no reference gene is truly stably expressed for any type of stress, although many studies are being conducted^37^. Differential expression of the internal gene under different experimental conditions or at different developmental stages causes errors in normalization of relative expression levels. Furthermore, the variability of the reference gene has been easily overlooked as an advantage of gene expression studies using this approach. This means that the use of a reference gene for calculating the expression of target genes under specific conditions is always required when new species are used in ecotoxicological studies^14^.

Reference gene validation has been performed in many other aquatic invertebrates, such as cladocera *Daphnia magna*^13^, amphipod *Gammarus fossarum*^14^, and bivalves *Pecten maximus* L.^38^ and *Ruditapes philippinarum*^39^. In particular, Volland et al.^39^ demonstrated that many invalidated uses of genes still introduce artefactual variance, causing misinterpretations of gene expression. These studies insist that preliminary investigations, such as conducting basic research to find reference genes, are important for studies using gene expression as well as for toxicogenomics. Furthermore, the results of studies on reference genes have been helpful in using these species in ecotoxicological studies, followed by the suggestion of specific genes^40,41^. In the brackish water flea, *D. celebensis* is also one of the potential species that might be used in several studies on pollution in many tropical and coastal areas, either in lab-based work or fields^26,42,43^. The nine candidate reference genes, *Atb, Act, 18S, GAPDH, EF-1b, UBC, H2A, TBP*, and *SDH* were used to identify adequate and reliable reference genes for brackish water flea exposed to waterborne chemicals. In our study, however, no reference gene was excluded from analysis because all primer pairs yielded sufficient efficiencies in RT-qPCR (Ct values < 30).

In the four different approaches for stability testing, each method selected the stability of the potential reference genes with different orders (Table S1). When considering all samples for the three chemical exposures, NormFinder showed different results in the order (*H2A*, *Act*, *EF-1b*, *18S*) of stability compared to those collected by geNorm (*H2A*, *EF-1b,* and *TBP*). Many studies insist that these two methods yield similar results, but the discrepancy between geNorm and NormFinder in this study might be explained by the high expression shown in *18S* (approximately two-fold Ct value smaller than any other gene). The BestKeeper, which indicates the standard deviation (SD) of Ct is considered stable when a value less than 1 leads to similar results from NormFinder for the two most stable genes.

To validate the effect of the reference gene, we compared the target genes under different conditions of the reference gene. *GST*, selected as a target gene, is a phase II detoxifying enzyme that involves the removal of electrophilic substances using reduced glutathione (GSH) as a substrate in most organisms^44^. In particular, it is well established as a biomarker for diagnosis of pollution as GST mediates the binding between reactive metabolites generated by phase I enzymes and the thiol group of GSH, thereby converting it to a nontoxic form^45^. Our recent study also showed that the GST isoform, particularly *GSTmu* of *D. celebensis* normalized by *18S rRNA*, was similarly upregulated in the presence of heavy metals and BPA exposure^47,48^. The increased patterns of *GSTmu* observed in the chemical exposures demonstrated that the calculated results based on reference genes in this study are reasonable. Furthermore, the results in which chemical exposures did not elicit comparable patterns in target genes suggested that all reference genes could be candidates when using individuals at least by day 4 of these three chemical exposures (Fig. 4). These results were expected, as all genes used in this batch had M values at geNorm significantly lower than the cut-off, 1.5 (Fig. 1A).

On the other hand, the different ages cause differential expression of reference genes, even though these nine genes are believed to be suitable for reference and showed stable results in other studies, including our first batch of experiments conducted under chemical exposure^27–30^. First, the alterations of *GSTsigma* at different ages suggested that age may have different abilities to metabolize, resulting in different gene expressions. In *D. celebensis*, 4-day old release the first brood of neonates^23^ and 7-day old are in the molting period (unpublished data). This indicates that different age groups may lead to large alterations in the expression of some reference genes. As expected, the result calculated from the use of an unstable reference (*Atb*) causes a distinct pattern of *GSTsigma*, as if the 4-day individuals were overestimated.

The expression patterns of another selected target gene, ecdysteroid receptor A (*EcRA*), was also similar to *GSTsigma*. EcRA is a key marker that is involved in the transport of ecdysteroid, a molting hormone in arthropods, into the nucleus, and thus plays a key role in the modulation of growth, reproduction, and development^49^. Differently modulated expression according to age with the molting process suggested that these genes can be markers of *D. celebensis* to evaluate development^21^. However, the selection of *Atb* resulted in significantly different results in target gene expression. This indicates that the Ct value of *Atb* highly fluctuates according to age. This can be a falsification to report that *Atb* levels remain stable and good candidates as a reference gene for this organism as day 4 is the tipping point of life table for the growth of *D. celebensis*. In the case of both *GAPDH* and *Act*, there was no difference in the expression level in each group, but the expression level of target genes was significantly upregulated in a normalization with *Atb* (t-test, *p* < 0.05). Previous studies have also pointed out that some reference genes can be differently suggested according to the developmental stage of the plant^50,51^. Thus, the results obtained from *D. celebensis* show evidence of the importance of reference gene selection to avoid misinterpretation in specific organisms that have distinctive growth and reproductive periods in life tables.

In addition, the *18S rRNA* gene was classified as stable by the four different algorithms despite showing a significantly lower Ct value. This is interesting, as *18S rRNA* has been considered to be one of most popular reference genes, as it is expressed in most cell types, but several identified problems suggest that *18S rRNA* may lead to unexpected analytical errors in the cDNA synthesis step, and the ribosomal fraction may not actually represent the whole cellular mRNA population^52^. These results suggest that additional research and validation under various environmental and physiological conditions are required to validate the use of *18s rRNA*.

## Conclusion

In our study, nine candidate reference genes were selected for real-time qRT-PCR standardization and evaluated using four different algorithms. The different analytical methods result in comparable values and ranks of the candidates, but the integrated application of these results leads to more reliable information on which gene must be sorted out. The results showed that *H2A* and *Act* were the most stable reference genes for considering 4th day individuals of brackish water flea for chemical exposure, while *Atb* was the most unstable reference gene.

The results for the unstable gene were the same in terms of age. However, the selection of the reference gene results in a significantly different pattern in the target gene when prominent development or alterations, such as molting or reproduction, are observed. This is of great importance in marine environments, as most hypotrophic organisms have a short life cycle, rapid sexual maturation, and prominent features such as idiosyncratic growth, such as molting.

The stable reference genes identified in this study will enhance the accuracy of qRT-PCR-based analysis of target gene expression and can be used to study related ecotoxicological applications using this species in estuarine environments. In this study, normalization with the most stable or with a combination of two carefully chosen reference genes led to different patterns in the expressions that were calculated using unsuitable reference genes. Thus, careful evaluation of the reference genes prior to expression studies is essential. For future directions in environmental studies, more studies are required as the selection of appropriate reference genes from field conditions might be more complex than controlled environmental studies. However, the results accumulated in many studies will be useful for extracting candidates for future studies.

## Materials and methods

### Reagents

All chemical reagents used in this study are listed in Table S3. Briefly, all reagents for molecular studies and chemical exposure were of ultrapure grade for molecular biology and analytical grade (HPLC grade). Oligonucleotide synthesis and DNA sequencing analyses were performed by Bionics Co., Ltd. (Seoul, South Korea).

### Culture and maintenance

The cladoceran *D. celebensis* strain was maintained at the Molecular Toxicology Laboratory of Sangmyung University, South Korea, since they were obtained from Dr. Kyun-Woo Lee of the Korea Institute of Ocean Science & Technology (KIOST; Busan, South Korea) in 2015. The culture medium used was 0.2 μm-filtered artificial seawater (15 psu) using Instant Ocean (Aquarium system, France). Culture conditions were maintained at a photoperiod of 12 h:12 h light/dark and 25 ± 1 °C. The food source was *Chlorella vulgaris* (4 × 10^7^ cells/L) cultured in Jaworski’s medium and supplied once every 2 days.

### Waterborne exposure to chemicals

Three chemicals (BPA, B[a]P, and Hg), which have been extensively studied in aquatic environments, were selected as representative toxicants. Stock solutions of BPA (2,2,-bis-(4-hydroxyphenyl) propane; 3 mg/ml), B[a]P (5 mg/ml) were prepared by dissolving in dimethyl sulfoxide (DMSO), and those of Hg (HgCl_2_) were dissolved in distilled water at a concentration of 1 mg/ml. Working solutions were prepared by adding stock solution to 200 ml of 15 psu sea water. To examine the concentration-dependent effects of chemicals, *D. celebensis* (4 days old, ~ 200 individuals/each concentration) was exposed to BPA (1.5, 3, and 6 mg/L), B[a]P (0.3, 0.6 and 1.2 mg/L), and Hg (2.5, 5, and 10 μg/L) for 48 h in a 250 ml beaker. The concentrations of each chemical were designed based on an acute toxicity test that was conducted as a preliminary study (Table S2). In addition, to check the developmental stage-dependent effects, 24 h, 4 d, 7 d, and 10 d old *D. celebensis* (~ 200 individuals/each concentration) were used. The final DMSO concentration was less than 0.05%, and no mortality was observed. All examinations were performed in triplicates.

### Total RNA extraction and cDNA synthesis

*D. celebensis* was collected 48 h after exposure to chemicals or at different developmental stages and homogenized in five volumes of TRIzol reagent (Thermo Fisher Scientific Inc., USA). Total RNA was extracted according to the manufacturer’s instructions and kept at -80 °C until use. The quality and quantity of total RNA were checked using agarose gel electrophoresis and a Nano drop (Maestrogen Nano Drop, Taiwan). Only RNA samples with a 260/280 nm ratio of 1.8–2.0 were used. cDNA was synthesized from 500 ng of total RNA using the RevertAid First Strand cDNA Synthesis Kit (Thermo Fisher Scientific Inc., USA).

### Polymerase chain reaction (PCR) amplification and sequence analysis

The cDNA sequences of nine reference genes (*Atb*, *Act*, *18S*, *GAPDH*, *EF-1b*, *UBC*, *H2A*, *TBP*, and *SDH*) were obtained from the local database of *D. celebensis* transcriptome (Molecular Toxicology Laboratory, Sangmyung University). The PCR protocol was as follows: 95 °C for 2 min; 35 cycles of 95 °C for 30 s, 55 °C for 30 s, 72 °C for 2 min; and an extension step at 72 °C for 10 min. The PCR product was visualized on a 1.4% agarose gel and purified using the AccuPrep ® Gel Purification Kit (Bioneer, South Korea) for sequencing. Basic Local Alignment Search Tool (BLAST) homology searches of databases were performed using Blast + 2.8.1 from the NCBI to identify the gene. The sequences of each gene were deposited in GenBank (Table 1).

### Quantitative real-time RT-PCR

To validate the reference genes, real-time qRT-PCR was performed using a CFX96™ real-time PCR system (Bio-Rad, USA). Each PCR reaction involved 2 μl cDNA and 2 μl of 10 pmol primer set (Table 1). The PCR cycle conditions were as follows: 95 °C for 10 min, followed by 33 cycles of 95 °C for 15 s, 60 °C for 1 min. To check a specific product after PCR, melting curves were analyzed under the following conditions: 95 °C for 15 s and 60 °C for 1 min with a 0.5 °C increase per second (Text S1). All experiments used SYBR master mix (KAPA Bioassay System, USA) and performed in triplicate. The threshold cycle (Ct) from each experiment was used to compare the stability of the reference genes. All examinations were performed in triplicates.

### Expression stability analysis of candidate reference genes

The Ct values obtained from qRT-PCR were analyzed using geNorm (Vandesompele et al. 2002), NormFinder^55^, BestKeeper^56^, and RefFinder (https://www.heartcure.com.au/reffinder/) algorithms. For geNorm, the expression stability of nine reference genes was evaluated using statistical analysis and the average expression stability (M value) using geNorm analysis^54^ in qbase PLUS 3.2 (Biogazelle, Ghent, Belgium). Similar to geNorm, NormFinder also calculated the average expression stability values based on the 2^−∆Ct^ value, and low values were stable. BestKeeper, a method using the Ct value directly without any conversion step, showed stability based on the coefficient of variance (CV) and standard deviation (SD), and RefFinder integrating all the results from the three software mentioned above, was used to calculate the ranking index for showing stability in this study. In addition, the optimal number of reference genes required for reliable normalization was also evaluated by pairwise variation (V value) through geNorm analysis^54^.

### Normalization with reference genes

From the stability analysis, the less stable and the most stable reference genes were used to normalize the expression of one target gene (glutathione S-transferase mu, *gst-mu*) in 4-day old *D. celebensis* exposed to chemicals for 48 h. On the other hand, one less stable and two more stable reference genes were used to normalize the expression of two target genes (ecdysone receptor A, *EcRA*; glutathione S-transferase, *GST-sigma*, and *GST-mu*) in *D. celebensis* of four different ages. These genes were selected as target genes because they showed the most significant changes under chemical exposure conditions in our previous studies^44,47–49^. The fold change values were calculated using the 2^△△Ct^ method^57,58^ using formulas (1) and (2):

1 $${\text{Average}}\,{\text{CT}}\,{\text{of}}\,{\text{reference}}\,{\text{genes}}\,\left( {{\text{RG}}} \right):\,{\text{CT}}_{{{\text{RG}}}} \, = \,\left( {{\text{CT}}_{{{\text{RG1}}}} \, + \,{\text{CT}}_{{{\text{RG2}}}} \, + \cdots + \,{\text{CT}}_{{{\text{RGn}}}} } \right)/{\text{n}}$$

2 $${\text{Gene}}\,{\text{expression}}\,{\text{level}}:\,{\text{G}}\, = \,{2}^{{ - \, ({\text{CTgene }}{-}{\text{ CTRG}})}}$$

### Statistical analysis

Data from all experiments are represented as the mean ± standard deviation (SD) of the three replicates. One-way analysis of variance (one-way ANOVA) and Student’s t-test followed by Tukey’s test were used for statistical analysis among samples using the PASW statistics 18.0 program (SPSS Inc., Chicago, IL, USA). Statistical significance was set at *p* < 0.05.

## Supplementary Information

Supplementary Information.
