# Supplementary material for: Validation of reference genes for quantitative real-time PCR in chemical exposed and at different age’s brackish water flea Diaphanosoma celebensis
Source: Sci Rep. 2021 Dec 8;11:23691. doi: 10.1038/s41598-021-03098-x (PMC8654955; doi:10.1038/s41598-021-03098-x)
Supplement: Supplementary file 1 — Supplementary Information. [file 41598_2021_3098_MOESM1_ESM.docx]

**<Supplementary Information>**

**Validation of reference genes for quantitative real-time PCR in chemical exposed and at different age’s brackish water flea *Diaphanosoma celebensis***

Young-Mi Lee^1,*^, Hayoung Cho^1^, Ryeo-Ok, Kim^1,†^, Soyeon In^1^, Se-Joo Kim^2^, Eun-Ji Won ^3*^

*^1^Department of Life Science, College of Natural Sciences, Sangmyung University, Seoul 03016, Republic of Korea*

*^2^* *Genome Editing Research Center, Korea Research Institute Bioscience and Biotechnology, Daejeon, 34141, Republic of Korea*

*^3^* *Department of* *Marine Science and Convergent Technology, Hanyang University, Ansan 15588, Republic of Korea*

*^4^* *Institute of Marine and Atmospheric Sciences, Hanyang University, Ansan 15588, Republic of Korea*

Number of pages: 17 including cover and contents

Number of texts: 2

Number of tables: 3

Number of figures: 10

**Contents**

**Supplementary Text**

Text S1. Verification procedures for amplified products of each gene S4

Text S2. Standard curves for determination of primer pair efficiency S5

**Supplementary Tables**

Table S1. Comprehensive ranking of reference genes according to four different algorithms for stability test (geNorm, NormFinder, BestKeeper, and RefFinder) S6

Table S2. Preliminary acute toxicity tests for selecting the concentration ranges of chemicals S7

Table S3. Chemical information used in this study S8

**Supplementary Figures**

Figure S1. Box-and whisker plot of nine potent reference genes in samples from two experimental conditions, A) chemical exposure and B) developmental stages. S9

Figure S2. Average expression stability values (M) and ranking of the candidate reference genes calculated using geNorm according to different chemicals. (A) BaP, (B) BPA, (C) Hg, and (D) entire samples exposed to each chemical S10

Figure S3. Average expression stability values and ranking of the candidate reference genes calculated using NormFinder according to different chemicals. (A) BaP, (B) BPA, (C) Hg, and (D) entire samples exposed to each chemical S11

Figure S4. Average expression stability values (coefficient, r) and ranking of the candidate reference genes calculated using BestKeeper according to different chemicals. (A) BaP, (B) BPA, (C) Hg, and (D) entire samples exposed to each chemical S12

Figure S5. Average expression stability ranking of the candidate reference genes integrated by RefFinder according to different chemicals. (A) BaP, (B) BPA, (C) Hg, and (D) entire samples exposed to each chemical S13

Figure S6. Average expression stability values (M) and ranking of the candidate reference genes calculated using geNorm according to different developmental stage. (A) 24hs, (B) 4 days, (C) 7 days, (D) 10 days and (D) entire samples of all development stages S14

Figure S7. Average expression stability values of the candidate reference genes calculated using NormFinder according to different developmental stage. (A) 24hs, (B) 4 days, (C) 7 days, (D) 10 days and (D) entire samples of all development stages S15

Figure S8. Average expression stability values of the candidate reference genes calculated using BestKeeper according to different developmental stage. (A) 24hs, (B) 4 days, (C) 7 days, (D) 10 days and (D) entire samples of all development stages S16

Figure S9. Average expression stability ranking of the candidate reference genes integrated by RefFinder according to different developmental stage. (A) 24 h, (B) 4 d, (C) 7 d, (D) 10 d and (D) entire samples of all development stages S17

Figure S10. The relative expression level of *GSTsigma* and *EcRA* determined using each candidate reference gene in the brackish water flea *D. celebensis*. Each bar indicates different ages (12 h, 4 d, 7 d, and 10 d-post hatching). Capital and small letters indicate significant differences by age and genes used for normalization, respectively. (ANOVA, Tukey’s post-hoc analysis, *p*<0.05). S18

**Supplementary Text**

**Text S1.** Verification procedures for amplified products of each gene.

First, the unimodal melting peak of each target gene was checked in program for verifying the primer specificity (Image 1). Additionally, the amplified PCR product was analyzed on a 1% agarose gel under UV transilluminator to check the single band for confirming the specific amplification of target genes. Then, the sequencing of the PCR product was conducted for further verification of the product (Macrogen, Seoul, Korea)

**Image 1**. Melting curves of each potential reference gene targeted in this study. A) *Alpha-tubulin* (*Atb*), B) *beta-actin* (*Act*), C) *18s ribosomal RNA* (*18s*), D) *Glyceraldehyde-3-phosphate dehydrogenase* (*GAPDH*), E) *Elongation factor 1-bata* (*EF-1b*), F) *Ubiquitin conjugating enzyme* (*UBC*), G) *Histone H2A* (*H2A*), H) *TATA-box binding protein* (*TBP*), I) *Succinate dehydrogenase* (*SDH*)

1. B) C)


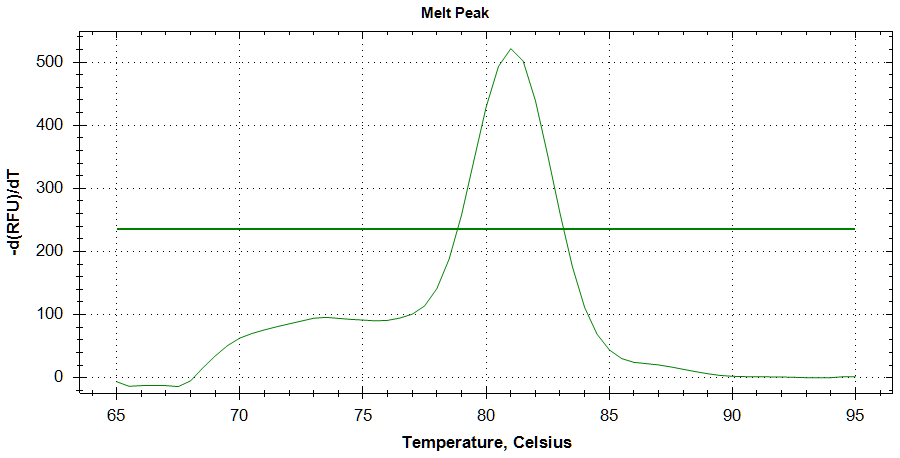

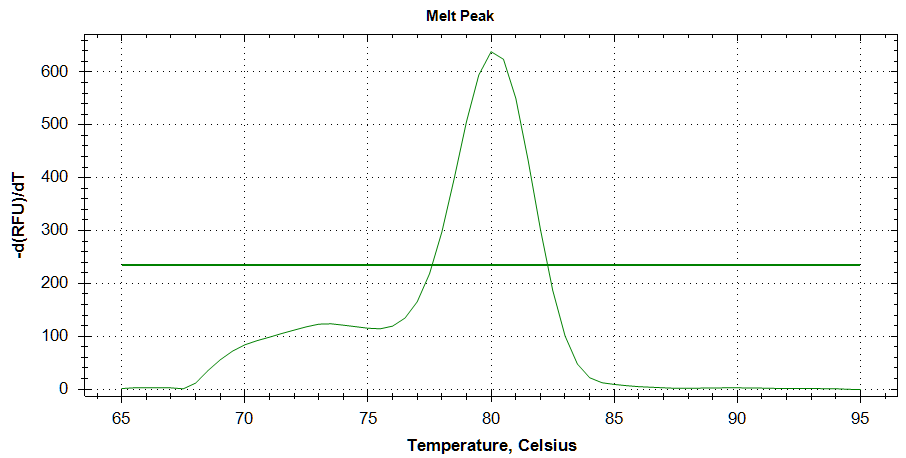

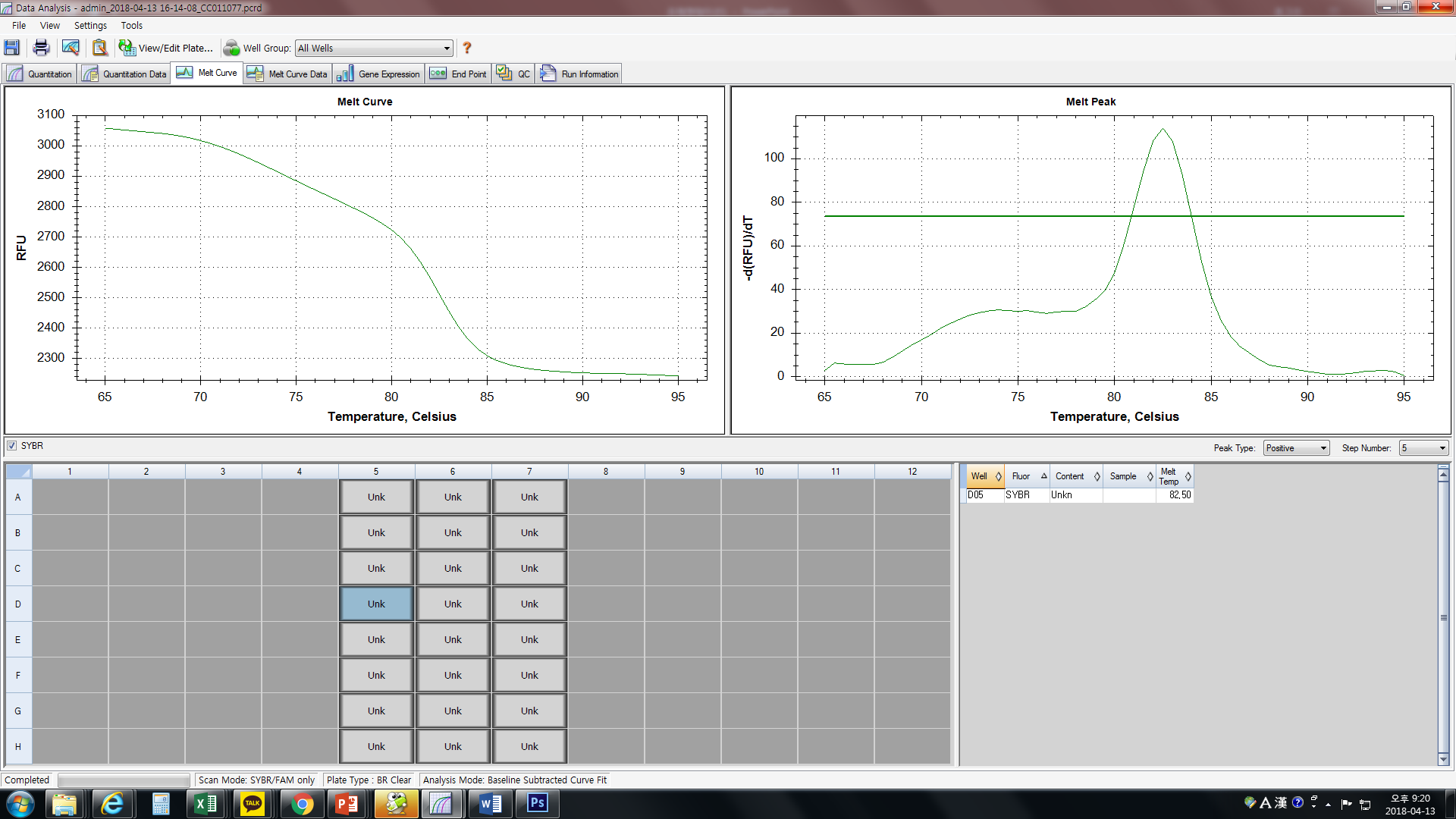


D) E) F)


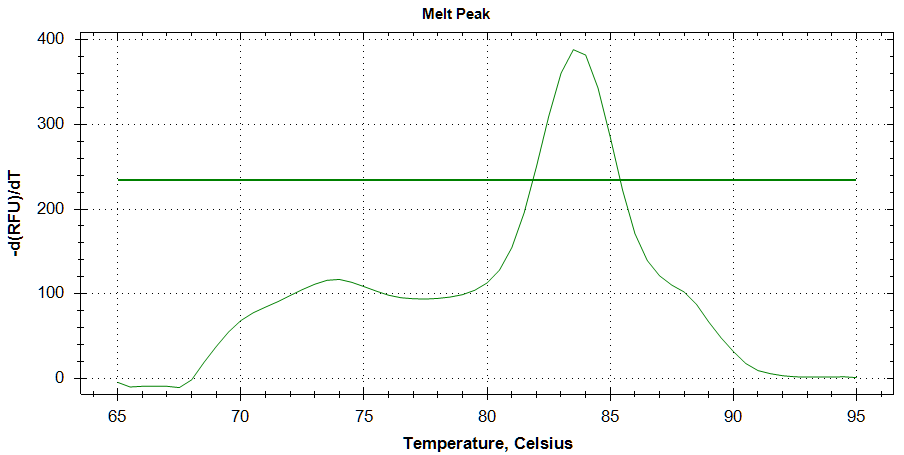

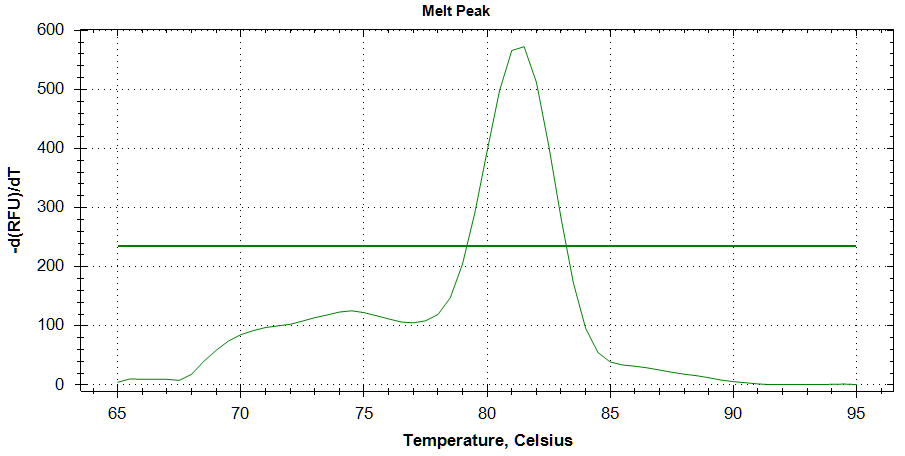

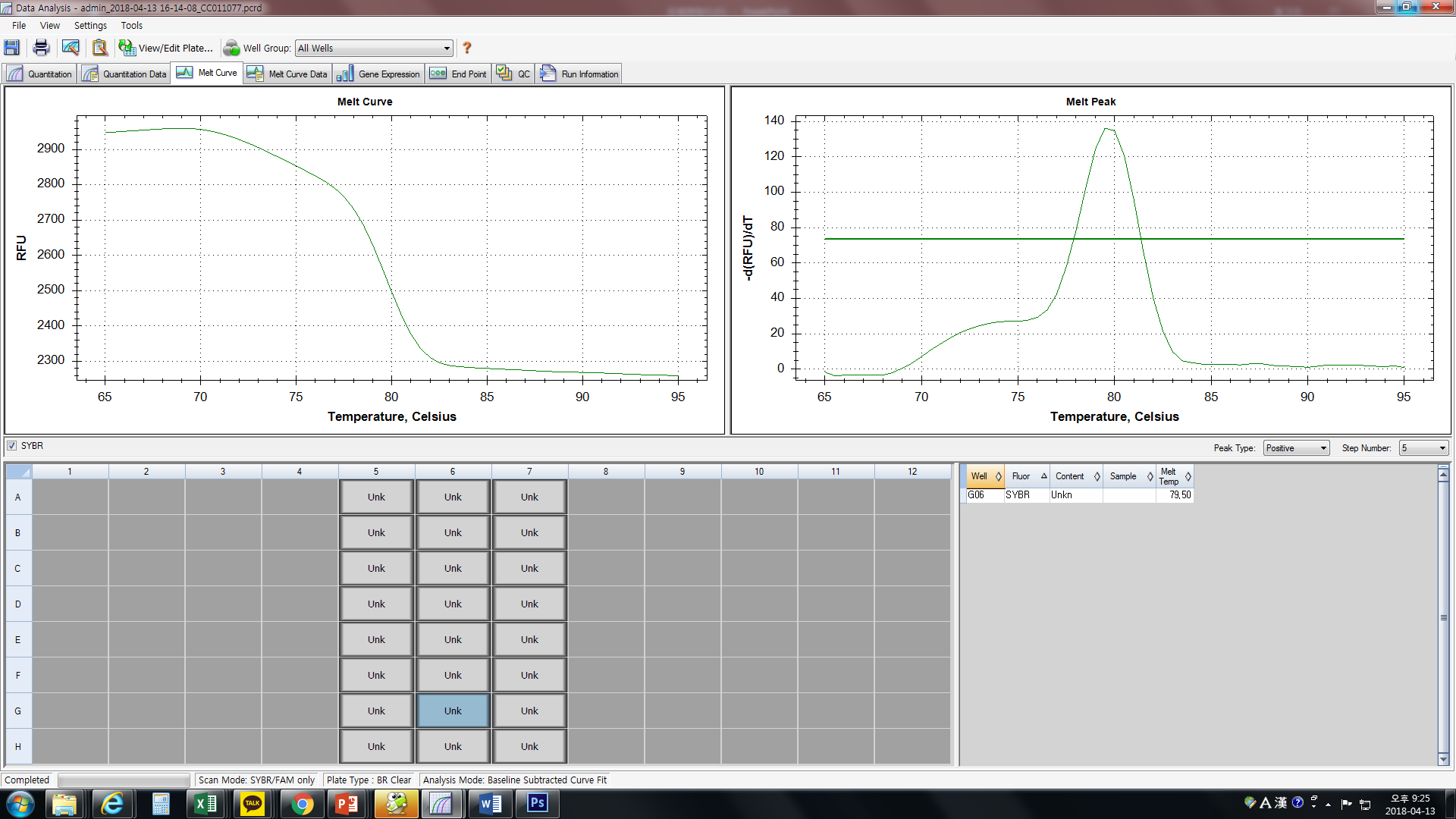


G) H) I)


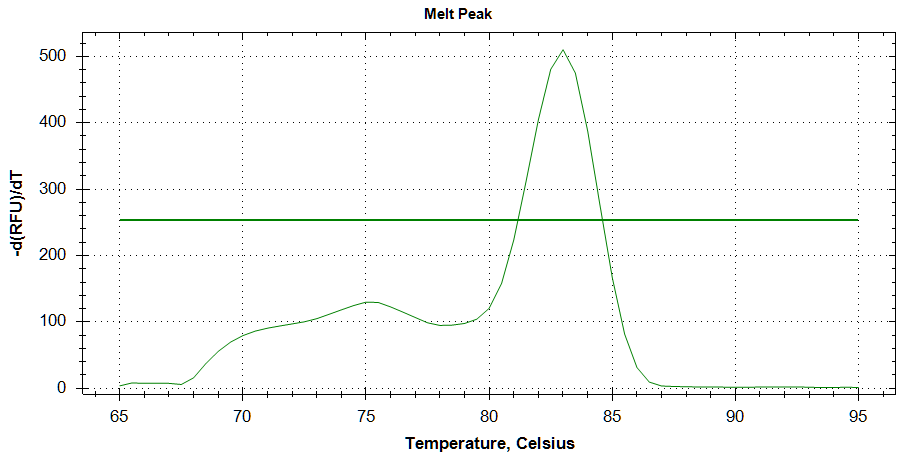

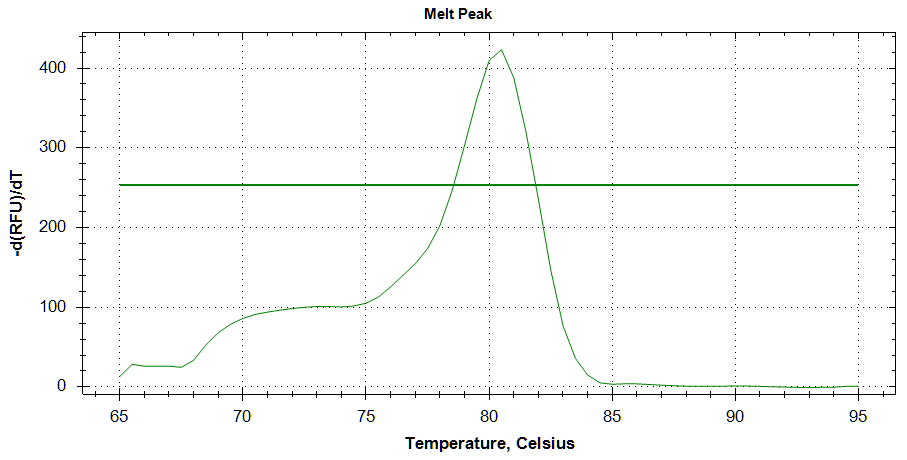

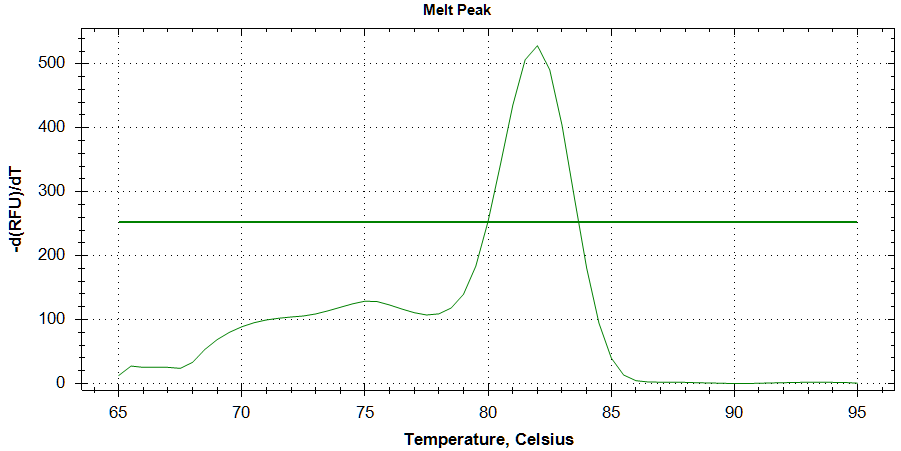


**Text S2.** Standard curves for determination of primer pair efficiency.

For the generation of standard curves all PCR-insert-containing TOPO constructs were pooled by mixing 10^10^ copies of each plasmid. From this pool, serial dilutions (containing10^8^-10^2^ copies per PCR-reaction) were used for qPCR with each primer pair. The PCR amplification efficiency of each primer pair was calculated using the equation:

Efficiency% = (10^[-1/slope]^ -1) x 100%.

**Table S1.** Comprehensive ranking of reference genes according to four different algorithms for stability tests. Two lines, Chemical exposure and ages represent different test groups to validate the stability of the candidate gene. B[a]P, BPA, and Hg were used for chemicals exposure tests. Four groups in a different age (12h, 4 day, 7 days, and 10 days post hatching) were used for another test.

|  | **Chemical exposure** | | | |
| --- | --- | --- | --- | --- |
| **Rank** | **geNorm** | **NormFinder** | **BKSTD** | **RefFinder** |
| 1 | *EF-1b/ H2A* | *H2A* | *Act* | *H2A* |
| 2 |  | *Act* | *H2A* | *Act* |
| 3 | *TBP* | *EF-1b* | *SDH* | *EF-1b* |
| 4 | *UBC* | *18S* | *EF-1b* | *TBP* |
| 5 | *Act* | *UBC* | *TBP* | *18S* |
| 6 | *18S* | *TBP* | *18S* | *UBC* |
| 7 | *SDH* | *SDH* | *GAPDH* | *SDH* |
| 8 | *GAPDH* | *GAPDH* | *UBC* | *GAPDH* |
| 9 | *Atb* | *Atb* | *Atb* | *Atb* |
|  | **Ages** | | | |
| 1 | *Act/ GAPDH* | *GAPDH* | *SDH* | *Act* |
| 2 |  | *EF-1b* | *H2A* | *GAPDH* |
| 3 | *EF-1b* | *Act* | *Act* | *SDH* |
| 4 | *SDH* | *18S* | *UBC* | *EF-1b* |
| 5 | *H2A* | *H2A* | *GAPDH* | *H2A* |
| 6 | *UBC* | *SDH* | *EF-1b* | *UBC* |
| 7 | *TBP* | *UBC* | *TBP* | *18S* |
| 8 | *18S* | *TBP* | *18S* | *TBP* |
| 9 | *Atb* | *Atb* | *Atb* | *Atb* |

Table S2. Preliminary acute toxicity tests for selecting the concentration ranges of chemicals.

|  | **EC50-24h (95% CI)** | **EC10-24h (95% CI)** | **NOEC** |
| --- | --- | --- | --- |
| B[a]P | 6.852 (0.022 – 2110.171) mg/L | 1.169 (0.112 – 12.161) mg/L | ≥ 0.600 mg/L |
| BPA | 7.454 (6.790 – 8.183) mg/L | 5.715 (4.923 – 6.635) mg/L | ≥ 15.00 mg/L |
| Hg | 0.013 (0.012 – 0.015) mg/L | - | - |

Table S3. Chemical information used in this study

| **Protocols** | **Compounds (or Kit)** | **Company** | **Catalog no.** | **Remarks** |
| --- | --- | --- | --- | --- |
| Chemical exposure | DMSO | Sigma-Aldrich | D8418 |  |
|  | B[a]P | Sigma-Aldrich | B1760 | ≥96% |
|  | BPA | Sigma-Aldrich | 239658 | ≥99% |
|  | Hg | Sigma-Aldrich | 215465 | ≥99.5% |
| RNA extraction | Trizol^TM^ Reagent | Thermo Fisher Scientic Inc. | 15596018 |  |
|  | Chloroform | Sigma-Aldrich | C2432 | ≥99.5% |
|  | Isopropyl alcohol | Sigma-Aldrich | I9030 | ≥99% |
|  | Ethyl alcohol | DUKSAN Reagent | D5 | 99.9% |
|  | DEPC-DW | Bioneer | C-9030 |  |
|  | Deionized sterile D.W | Bioneer | C-9011 |  |
| cDNA synthesis | RevertAid First Strand cDNA Synthesis Kit | Thermo Fisher Scientic Inc. | K1621 |  |
| Sequence analysis | AccuPrep® Gel purification Kit | Bioneer | K3038 |  |
| Quantitative real-time RT-PCR | KAPA SYBR Fast Qpcr Universal readymix kit | KAPA Bioassay System | KK4602 |  |

**Figure S1**. Box-and whisker plot indicates ranges of cycle threshold (Ct) values of nine potent reference genes in samples from two experimental conditions, A) chemical exposure and B) ages (days) of *D. celebensis*. The box indicates the Ct value within the 25^th^ and 75^th^ percentiles. Whiskers include the Ct values in the 10^th^ and 90^th^ percentiles. The line across the box represents the median.


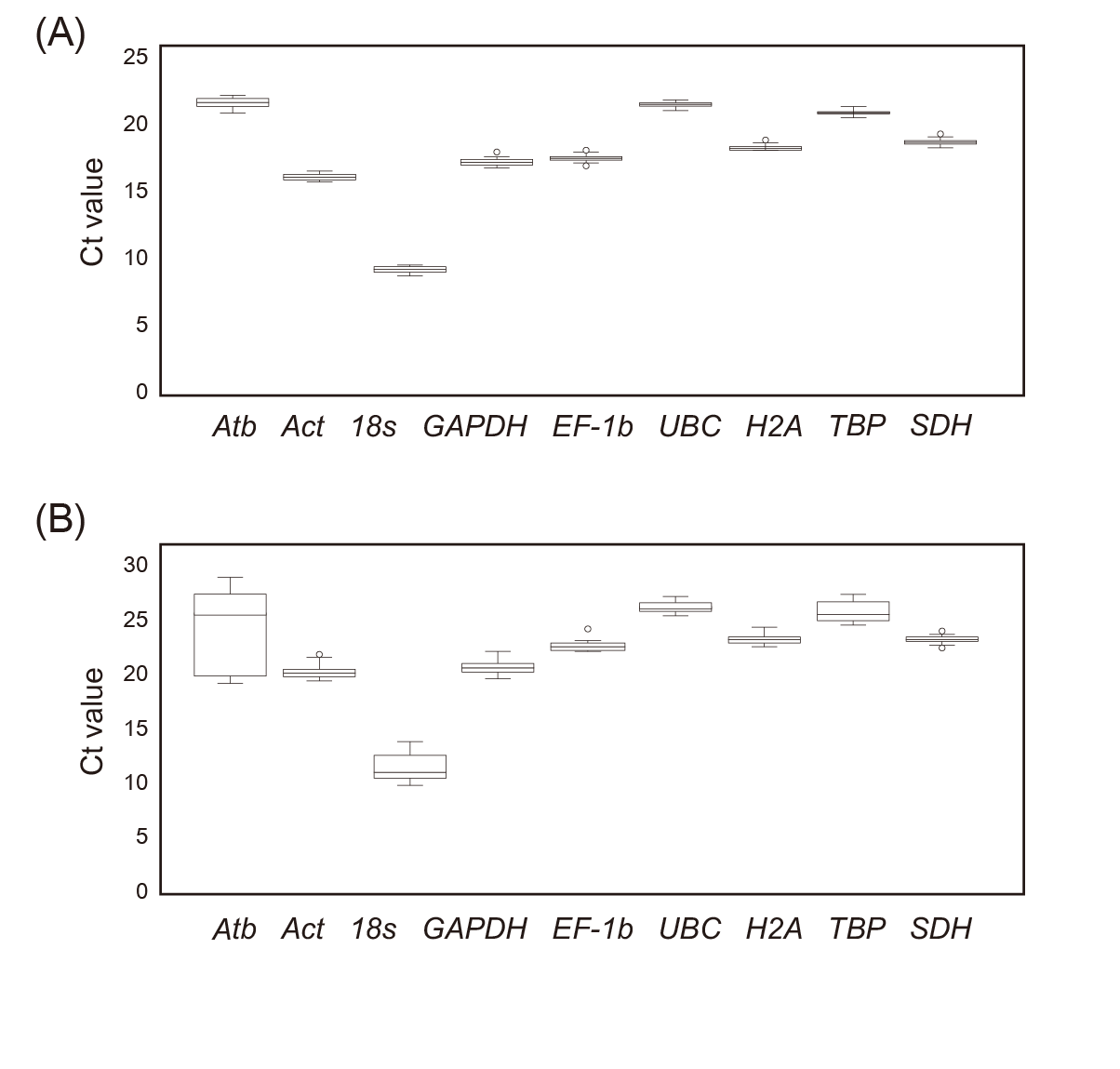


Figure S2. Average expression stability values (M) and ranking of the candidate reference genes calculated using geNorm according to different chemicals. (A) BaP, (B) BPA, (C) Hg, and (D) entire samples exposed to each chemical.

(A) (B)

(C) (D)

Figure S3. Average expression stability values and ranking of the candidate reference genes calculated using NormFinder according to different chemicals. (A) BaP, (B) BPA, (C) Hg, and (D) entire samples exposed to each chemical.

(A) (B)

(C) (D)

Figure S4. Average expression stability values (coefficient, r) and ranking of the candidate reference genes calculated using BestKeeper according to different chemicals. (A) BaP, (B) BPA, (C) Hg, and (D) entire samples exposed to each chemical.

(A) (B)

(C) (D)

Figure S5. Average expression stability ranking of the candidate reference genes integrated by RefFinder according to different chemicals. (A) BaP, (B) BPA, (C) Hg, and (D) entire samples exposed to each chemical.

(A) (B)

(C) (D)

**

Figure S6. Average expression stability values (M) and ranking of the candidate reference genes calculated using geNorm according to different developmental stage. (A) 24hs, (B) 4 days, (C) 7 days, (D) 10 days and (D) entire samples of all ages.

(A) (B)

(C) (D)

(E)

Figure S7. Average expression stability values of the candidate reference genes calculated using normFinder according to different developmental stage. (A) 24hs, (B) 4 days, (C) 7 days, (D) 10 days and (D) entire samples of all ages.

(A) (B)

(C) (D)

(E)

Figure S8. Average expression stability values of the candidate reference genes calculated using BestKeeper according to different developmental stage. (A) 24hs, (B) 4 days, (C) 7 days, (D) 10 days and (D) entire samples of all ages.

(A) (B)

(C) (D)

(E)

Figure S9. Average expression stability ranking of the candidate reference genes integrated by RefFinder according to different developmental stage; (A) 24hs, (B) 4 days, (C) 7 days, (D) 10 days and (D) entire samples of all ages.

(A) (B)

(C) (D)

(E)

Figure S10. The relative expression level of (A) *GSTsigma* and (B) *EcRA* determined using each candidate reference gene in the brackish water flea *D. celebensis*. Each bar indicates different ages (12h, 4d, 7d, and 10d-post hatching). Capital and small letters indicate significant differences by age and genes used for normalization, respectively (ANOVA, Tukey’s post-hoc analysis, p<0.05).

(A)

**
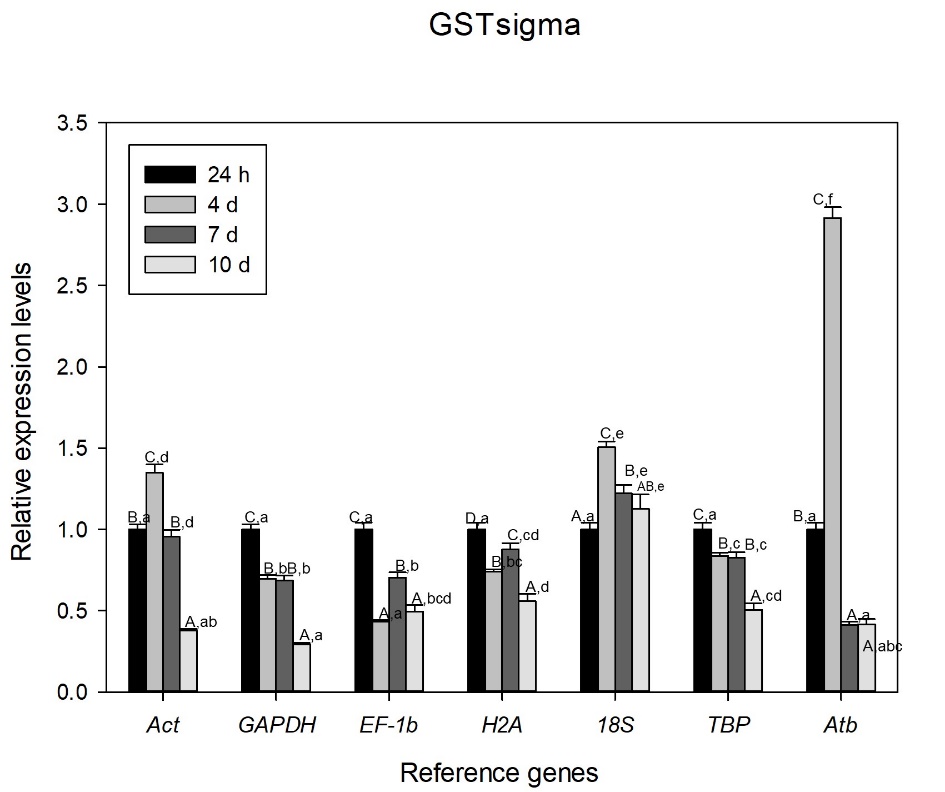
**

(B)

**
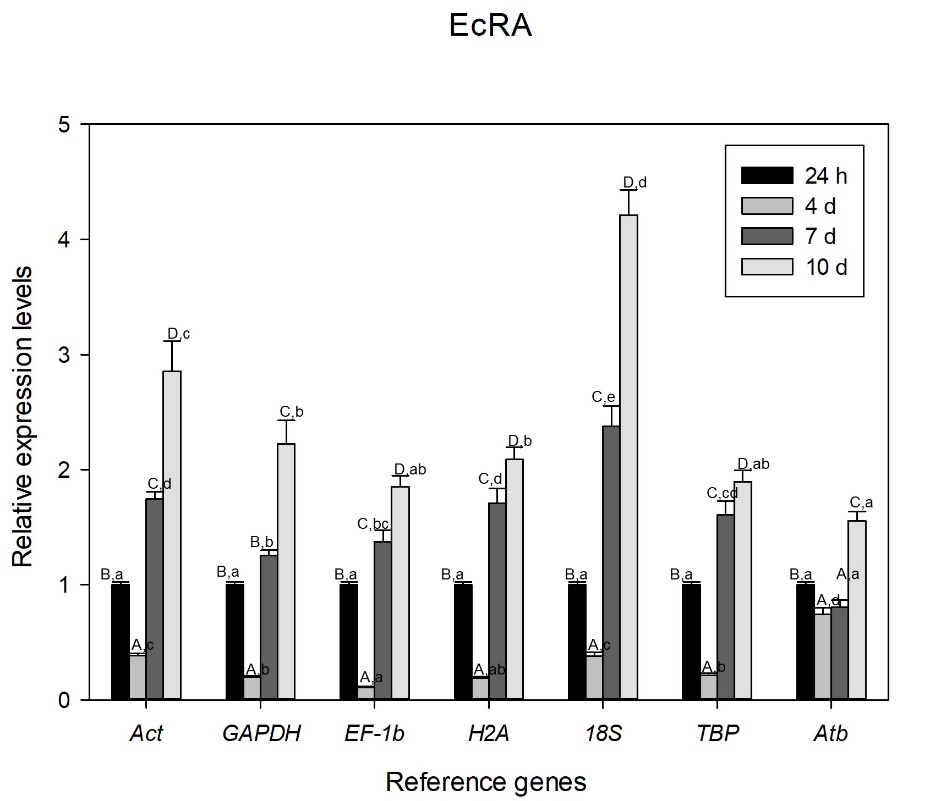
**
